# High resolution mapping of agro-morphological and grain traits in bread wheat using SNP-based QTL analysis

**DOI:** 10.1371/journal.pone.0340263

**Published:** 2026-01-02

**Authors:** Vasudha Jadon, Rahul Gajghate, Narayana Bhat Devate, Amaresh, Hari Krishna, Gopalareddy Krishnappa, Sweta Dosad, Neelu Jain, Pradeep Kumar Singh, Gyanendra Pratap Singh

**Affiliations:** 1 ICAR-Indian Agricultural Research Institute, New Delhi, India; 2 Protection of Plant Varieties and Farmers’ Rights Authority, Pusa, New Delhi, India; 3 ICAR-Sugarcane Breeding Institute, Coimbatore, India; 4 ICAR-National Institute of Seed Science & Technology, Bengaluru, India; 5 National Bureau of Plant Genetic Resources, New Delhi, India; Amity University, INDIA

## Abstract

Wheat (*Triticum aestivum* L.) is among the most extensively grown staple crops worldwide. A set of 188 recombinant inbred lines (RILs) derived from a cross between HD2932 and Synthetic 46 was evaluated over three consecutive years (2021–22, 2022–23, and 2023–24) for plant height (PH), spike length (SL), spikelets per spike (SPS), thousand kernel weight (TKW), kernel length (KL), kernel width (KW), and kernel thickness (KT). The population displayed wide phenotypic variability with quantitative inheritance for all the traits. High-density genotyping was performed using 910 SSR markers and a 35K SNP array. Twenty-eight QTLs, including six for PH, two for SL, three for SPS, two for TKW, five for KL, six for KW, and four for KT distributed across 16 chromosomes were identified. *QTkw.iari_4B*, flanked by *Xgwm149–AX-94559916*, was detected in all three environments (Q × E not formally tested) consistently and co-localized with QTLs for PH, KL, and KT, indicating a potentially important genomic region for yield improvement. Promising lines such as RIL 122 and RIL 66 exhibited superior kernel characteristics, while RIL 155 showed lower expression values. In silico analysis identified 28 candidate genes within these QTL regions, offering insights into trait regulation. These findings may serve as potential resources for marker-assisted selection in wheat breeding programs to enhance yield and grain quality parameters.

## Introduction

Wheat (*Triticum aestivum* L.) is one of the most important staple crops worldwide, providing approximately 20% of the calories to human’s diet [1,2]. Its significance in global food security is paramount, especially in the context of a rapidly growing population and the increasing challenges posed by climate change [3,4]. Enhancing wheat productivity and adaptability through genetic improvement remains a key objective in plant breeding programs. Yield and associated agro-morphological traits are polygenic and quantitative in nature, with complex inheritance and are often influenced by environmental interactions [5,6]. Therefore, dissecting the genetic architecture underlying these traits is crucial for effective crop improvement.

Molecular mapping, particularly quantitative trait loci (QTL) mapping, has become a valuable tool in modern wheat genetics, enabling identification of chromosomal regions controlling traits of agronomic importance [7,8]. Traditional QTL mapping relies on linkage maps developed from few distantly placed genetic markers, which track the inheritance of DNA polymorphisms in segregating populations. Over the last decade, advancements in high throughput genotyping technologies have facilitated the generation of high-density single nucleotide polymorphism (SNP) maps covering whole genome, providing unprecedented resolution for genetic analysis [1,9]. SNP markers are abundant, evenly distributed across the genome, and amenable to automated scoring, making them ideal for constructing detailed linkage maps [10]. High-density SNP based linkage maps allow precise localization of QTLs for complex traits, reducing confidence intervals and improving the accuracy of marker-assisted selection (MAS) [11,12]. Several studies have demonstrated the power of SNP arrays and genotyping-by-sequencing (GBS) in wheat for detecting QTLs associated with yield, disease resistance, and stress tolerance [13–16]. The integration of phenotypic data from well-characterised biparental populations with dense genotyping information enables robust mapping of complex traits. Agro-morphological traits such as plant height, spike length, tiller number, and kernel traits, along with yield components, are key determinants of wheat productivity [17–20]. These traits exhibit continuous variation and complex inheritance patterns, making traditional phenotypic selection challenging. Molecular mapping facilitates the identification of major and minor effect QTLs controlling these traits, allowing breeders to dissect their genetic basis and deploy favorable alleles through MAS or genomic selection (GS) approaches [21,22].

Despite numerous QTL mapping efforts in wheat, the highly polyploid and large genome (~17 Gb) of hexaploid wheat presents challenges in marker development and map construction [23]. However, recent advances in reference genome assemblies and SNP genotyping platforms have significantly enhanced the ability to generate high-density linkage maps [24]. These resources have accelerated the discovery of QTLs linked to yield and related traits, facilitating their deployment in breeding pipelines. Furthermore, understanding the genetic control of yield-related traits under diverse environmental conditions is crucial to develop wheat varieties resilient to abiotic stresses such as drought and heat, which are becoming more frequent due to climate change [25,26]. High-resolution SNP linkage maps facilitate the detection of environment-specific and stable QTLs, supporting breeding for broad adaptation [27,28]. In summary, molecular mapping using high-density SNP linkage maps represents a powerful approach to dissect the genetic complexity of agronomic traits in wheat.

Despite the availability of several QTL studies in wheat, no high-resolution mapping effort has been conducted in the HD2932 × Synthetic 46 population under the Northwestern Plain Zone (NWPZ) conditions. Moreover, QTLs consistently identified in multi-environments for kernel morphology and yield components, anchored to the IWGSC RefSeq v1.0 genome, remain largely uncharacterized in this background. Addressing this gap is essential for enabling precise marker deployment in breeding programs targeting this production environment.

While many previous wheat QTL studies rely on single-season data, low-density marker sets, or conventional parental lines, our work offers several novel aspects: (i) the HD2932 × Synthetic 46 cross introduces novel variation from a synthetic D-genome donor, widening allelic diversity; (ii) multi-year phenotyping over three consecutive seasons provides repeated detection information; (iii) integration of a high-density genotyping platform (35K SNP array) together with SSR markers to increase map resolution; (iv) a focused trait set combining plant architecture and detailed kernel morphology (TKW, KL, KW, KT), and (v) in-silico candidate gene mining within QTL intervals to provide initial functional leads. These combined features improve QTL resolution and enable identification of co-localized, potentially consistent loci that are directly more relevant for marker-assisted selection.

The current study aims to develop a high-density SNP based linkage map using a RIL population and conduct molecular mapping of key agro-morphological and yield traits. This research addresses the gap in precise localization of QTLs controlling yield components in wheat, which is vital for marker-assisted genetic improvement. By integrating comprehensive phenotypic evaluation with dense genotyping, we seek to identify major-effect QTLs that can be utilized in breeding programs targeting yield enhancement and adaptability. This study contributes to the growing knowledge by providing new insights into the genetic loci governing yield and related traits, which can accelerate wheat improvement efforts globally.

## Materials and methods

### Research material and field trials procedures

A set of 188 recombinant inbred lines (RILs) was developed for this study through a cross between the high-yielding Indian bread wheat variety HD2932 and a synthetic hexaploid wheat line, Synthetic 46 (genome composition: AABB, 2n = 28 + DD, 2n = 14). The synthetic line was created at CIMMYT, Mexico, by hybridizing the tetraploid species *Triticum turgidum* (AABB, 2n = 28) with the diploid progenitor *Aegilops tauschii* (syn. *Triticum tauschii*) (DD, 2n = 14). Synthetic wheats are particularly valuable because they introduce novel allelic variation from the D genome donor, thereby increasing recombination and enabling structural variations such as translocations and transpositions that are rare in natural bread wheat. This broadens the genetic base and provides new opportunities for stress tolerance, disease resistance, and grain quality improvement. In contrast, natural bread wheat contributes genetic gains in terms of yield stability and wide adaptation. These 188 RILs, along with their parental lines, were grown under irrigated conditions for evaluation. Field trials were conducted at the ICAR-Indian Agricultural Research Institute (IARI), New Delhi, India, representing the typical agro-climatic environment of the Northwestern Plain Zone (NWPZ). The field trials were conducted in a randomized complete block design with two replications. Each experimental unit comprised a plot of three rows, each one metre in length, with a row spacing of 25 cm. All entries could be accommodated within a uniformly managed field due to smaller blocks size. Trait measurements were recorded on a per-plot basis, and plot means were used for all statistical analyses.The RIL population was evaluated across the years during the *rabi* 2021−22 (E-I), 2022−23 (E-II), and 2023−24 (E-III) cropping seasons (Supplementary Table 1 in S1 File). The traits assessed included key agro-morphological characteristics such as plant height (PH), spike length (SL), and spikelets per spike (SLS), as well as kernel-related parameters including thousand kernel weight (TKW), kernel length (KL), kernel width (KW), and kernel thickness (KT). Wheat was sown under timely sown conditions from November 1–15 during each season.

### Field phenotyping

The evaluation of agronomic traits was performed following the protocols described by Liu et al. [19]. Data were collected for three key agro-morphological characteristics: PH, SL, and SLS. The PH was measured in centimeters as the distance from the soil surface to the tip of the main spike, excluding awns, for each individual plant. Similarly, SL was recorded in centimeters from the base to the tip of the main spike, again excluding awns. The SLS was obtained by directly counting the number of spikelets present on the main spike. For each trait, observations were made on five biological replicates within each replication plot, and the average values were used for subsequent statistical analysis. At physiological maturity, 25–30 spikes were randomly selected and manually harvested from each replication for further analysis. Kernel trait data were recorded on a subset of 50 grains from each of the 188 RILs as well as the two parental genotypes. Using Vernier calipers, KL, KW, and KT were precisely measured in millimeters. The TKW was determined by weighing 1,000 grains from each genotype. A numerical grain counter was employed to ensure accurate counting, and the total weight was measured using a precision balance.

### High-density genotyping

The genotyping information and linkage map employed in this research were sourced from the earlier publication by Gajghate [29]. Genomic DNA was extracted from 20–25 day old seedlings of both parental lines and the RILs (in F_8_/ E-II) using the CTAB extraction protocol [30]. Genotyping was carried out using hybridization-based markers, comprising a 35K SNP chip from the Axiom® Wheat Breeders’ Array and a set of SSR markers. SNP genotyping with the 35K Axiom® Wheat Breeder’s Array was performed using the Affymetrix GeneTitan® system, adhering to the manufacturer’s recommended protocol. Allele calling and genotype calling were executed with the Axiom Analysis Suite software, adhering to the Axiom® Best Practices Genotyping Workflow (Affymetrix user guide). A standard filtering protocol with minor allelic frequency less than 0.05 and call rate less than 0.9 (i.e., missing allelic frequency 0.1) were applied to get high quality SNP data.

For SSR marker analysis, primers from the Xwmc, Xgdm, Xbarc, Xgwm, Xcfa, and Xcfd series were selected based on the protocol outlined by Gajghate [29]. PCR amplification was performed in a 20 µL reaction mixture comprising 10 ng of genomic DNA, 5 pmol each of forward and reverse primers, 0.02 mM dNTPs, 0.3 units of Taq DNA polymerase (Bangalore Genie, Bengaluru, India), and 1X PCR buffer containing 16 mM MgCl_2_, 500 mM KCl, 100 mM Tris-HCl (pH 8.8), and 1% Triton X-100. The amplified PCR products were separated under low resolution conditions using either 3.5% agarose or 4% Metaphor agarose gels in TBE buffer, electrophoresed at 120 V for approximately 3 hours. The banding pattern of markers were scored. From the initial dataset of 35K SNP markers and 910 SSR markers, polymorphic loci differentiating the parental lines were first identified and grouped. A total of 836 robust markers—comprising 802 SNPs and 34 SSRs—were retained for linkage map construction. These markers were subsequently used to generate the linkage groups using the IciMapping software (version 4.2.53) [13]. Genetic distances, measured in centi Morgans (cM), were calculated using the Kosambi mapping function [31]. The final linkage map was visualized using the MG2C online platform (version 2.1) as described by Chao et al. [32].

### Statistical data analysis, mapping and identification of QTLs

Analysis of variance (ANOVA) and descriptive statistics were performed using the agricolae package in R and Microsoft Excel, respectively. A fixed effect model:


Yij=μ+Gi+Rj+ϵij

was used to for analysis of variance where, where $Y_{ij}$ denotes the observation of the i-th genotype in the j-th replication, μ is the overall mean, $G_{i}$ is the genotypic effect, $R_{j}$ is the replication (block) effect, and $\varepsilon_{ij}$ is the random error assumed to follow a normal distribution with mean zero and constant variance. Variance components such as phenotypic ($\sigma_{p}^{\text{2}}$) and genotypic variance ($\sigma_{g}^{\text{2}}$), coefficient of variation (CV) and heritability ($H^{\text{2}}$) were estimated from ANOVA model using following formula.


$$\sigma_{g}^{\text{2}}=\frac{{MSS}_{g}-{MSS}_{e}}{r}$$

Where, $\sigma_{g}^{\text{2}}$ is Genotypic variance, MSSg is genotypic mean sum of square, MSSr is error (residual) mean sum of square, r is the number of replications.


$$\sigma_{p}^{\text{2}}=\sigma_{g}^{\text{2}}+{MSS}_{e}$$

Where ${MSS}_{e}$ is error mean sum of square which is equal to error variance $(\sigma_{e}^{\text{2}}$). $\sigma_{p}^{\text{2}}$ is phenotypic variance. $\sigma_{g}^{\text{2}}$ is Genotypic variance.


$$PCV=\frac{\sqrt{\sigma_{p}^{\text{2}}}}{X}\times \text{1}\text{0}\text{0}$$

$$GCV=\frac{\sqrt{\sigma_{g}^{\text{2}}}}{X}\times \text{1}\text{0}\text{0}$$

$$H^{\text{2}}=\frac{\sigma_{g}^{\text{2}}}{\sigma_{p}^{\text{2}}}$$

Where, PCV is phenotypic coefficient of variance; GCV is genotypic coefficient of variance; X is the traits mean; H^2^ is the broad sense heritability.

Data visualization was performed through R-based tools such as ggplot2 for generating boxplots and frequency distributions, corrplot for constructing Pearson’s correlation matrices, and standard plotting functions from base R. QTL mapping was carried out using the Inclusive Composite Interval Mapping (ICIM) approach, implemented in IciMapping software (version 4.2.53, accessible at http://www.isbreeding.net). Both individual and combined phenotypic data across environments were integrated with the previously developed genetic linkage map for the identification of QTLs. Phenotypic records with missing values were excluded from the analysis. The QTL scan was executed with a step size of 1.0 cM, and the stepwise regression incorporated a selection threshold of p = 0.001. A manual LOD threshold of 2.5 was applied to identify significant QTLs. For each QTL detected, key information including the flanking molecular markers, genetic distance (in cM), LOD value, and the percentage of trait variance explained (PVE) was documented. QTL nomenclature followed the standard guidelines established by McIntosh et al. [33].

### In-Silico identification of candidate genes

To identify potential candidate genes associated with the detected QTLs, the nucleotide sequences of the significant SNPs and SSR markers flanking these loci were subjected to BLAST analysis using the Ensembl Plants database (http://plants.ensembl.org/Triticum_aestivum/Tools/Blast). The search was conducted using default parameters against the reference genome of bread wheat (*Triticum aestivum*), specifically the IWGSC RefSeq v1.0 assembly (2018) of the Chinese Spring variety. Candidate genes were explored within the QTL region itself as well as in a flanking interval of 0.5 Mb upstream of the right marker and 0.5 Mb downstream of the left marker to capture nearby functional loci. Gene functions were inferred based on prior research, focusing on their involvement in regulating agronomic and grain-related traits.

## Results

### Descriptive statistics, ANOVA, genetic parameters and correlation

The ANOVA across three environments (2021–22, 2022–23, and 2023–24) revealed highly significant differences among the RILs for all studied traits, indicating the presence of substantial genetic variability (Supplementary Table 2 in S1 File). Replication effects were significant for some traits such as plant height, kernel length, and kernel width, while error variances remained low, confirming the reliability of the data. The significant treatment effects across environments highlight the suitability of the population for identifying superior RILs and for subsequent QTL mapping of agronomic and grain quality traits. The parental genotype, Synthetic 46, consistently exhibited superior trait performance across all evaluated agro-morphological and kernel parameters in comparison to the high yielding Indian cultivar HD2932 (Table 1). A substantial degree of phenotypic variation was observed among the RILs across three environments for the agro-morphological traits SL, PH, and SPS with respective ranges of 73.3–166.6 cm, 7.79–17.79 cm, and 13.17–30.83. The mean values of promising RILs were compared against the parents’ average (HD 2932 and Synthetic 46), significant gains were observed for all the studied yield-contributing traits (Supplementary Table 3 in S1 File). For instance, RIL 73 exhibited remarkable improvement with 52.8% higher spike length, 31.4% more spikelets per spike, and 30.4% higher thousand kernel weight (TKW) compared to the parents’ mean. Similarly, RIL 118 also showed substantial gains with 44.2% higher spike length, 26.1% more spikelets per spike, and 26.9% higher TKW. RILs 28 and 90 likewise outperformed the parents, showing consistent improvements across spike length (29.2–37.9%), spikelets per spike (22.1–25.0%), and TKW (24.8–25.1%). Importantly, all these promising RILs showed enhanced kernel size parameters (length, width, and thickness) along with reduced plant height (18–27% lower than the parental mean), which is a desirable combination for yield stability and lodging resistance. Among these, percentage coefficient of variation (%CV) was under acceptable limits for all the traits under study. Broad-sense heritability estimates were highest for PH with 96.77%, followed by SL and SPS in E-I, indicating the presence of strong genetic control of these traits. Similar patterns were also observed for genotypic (GCV) and phenotypic (PCV) coefficients of variation. Conversely, environmental coefficient of variation (ECV) values exhibited a reverse trend, reflecting lower environmental influence on traits with high heritability. The highest estimates of genetic advance for all three traits were observed in the E-I, followed by E-III and E-II, suggesting substantial potential for selection gains.

**Table 1:** Descriptive statistics and genetic parameters of parental genotypes and recombinant inbred lines (RILs) evaluated across three years for agronomic traits: plant height (PH), spike length (SL), spikelets per spike (SPS); and kernel traits: thousand kernel weight (TKW), kernel length (KL), kernel width (KW), and kernel thickness (KT).

Trait	Parental Genotype Mean			Recombinant Inbred Lines (RILs)									
	Season	HD 2932	Syn46	Generation	Range	Mean	CV (%)	SE.d	H^2^(BS)	GCV	PCV	ECV	GA
PH	E-I	97.5	117.2	F7	73.3-148.8	75.5	2.39	2.53	96.77	13.13	13.35	2.39	28.22
	E-II	102.7	137.1	F8	80.5-162.2	120.9	6.7	8.10	78.86	12.94	14.57	6.70	28.64
	E-III	99	127.1	F9	91.8-166.6	129.9	7.12	9.24	70.55	11.017	13.11	7.11	24.76
SL	E-I	10.15	13.12	F7	8.65-15.62	11.42	3.95	0.451	89.35	11.44	12.11	3.95	2.54
	E-II	10.11	14.28	F8	8.5-15.52	11.59	8.85	1.02	51.86	9.18	12.75	8.85	1.57
	E-III	9.56	13.19	F9	7.79-17.69	10.64	6.15	11.3	61.32	11.68	10.58	10.90	3.31
SPS	E-I	20.01	17.66	F7	13.61-25.76	18.94	6.19	1.17	77.02	11.33	12.9	6.19	3.88
	E-II	19.69	17.33	F8	13.17-30.83	18.80	9.5	1.78	52.21	9.93	13.75	9.50	2.78
	E-III	20.7	20.01	F9	14.5-23.6	19.15	9.77	1.85	71.14	4.44	10.73	9.76	2.72
KL	E-I	6.28	8.24	F7	5.38-8.27	6.62	5.24	0.245	60.02	6.42	8.29	5.24	0.68
	E-II	6.17	7.21	F8	5.13-7.5	6.62	6.7	0.224	77.65	6.30	7.15	3.38	0.76
	E-III	6.07	7.02	F9	5.6-7.26	6.52	5.0	0.281	51.14	4.40	6.16	4.31	0.42
KW	E-I	3.50	2.98	F7	2.77-3.62	3.32	4.14	0.133	54.47	4.53	6.14	4.14	0.22
	E-II	3.50	3.04	F8	2.79-3.68	3.30	5.3	0.14	51.26	4.32	6.04	4.21	0.21
	E-III	3.31	2.97	F9	2.26-3.98	3.26	9.0	0.145	66.57	6.23	7.64	4.41	0.34
KT	E-I	2.88	3.60	F7	2.55-3.66	3.19	4.40	0.140	60.58	5.44	6.99	4.39	0.28
	E-II	2.68	3.26	F8	2.60-3.41	2.99	5.9	0.099	72.51	5.39	6.33	3.32	0.28
	E-III	2.84	3.38	F9	2.57-3.39	2.99	6.5	0.109	65.59	5.08	6.27	3.68	0.25
TKW	E-I	34.01	43	F7	21.12-51.76	37.42	3.80	1.42	93.96	14.98	15.45	3.79	11.19
	E-II	34.9	46.2	F8	25.2-51.4	40.67	12.3	6.54	65.96	7.072	17.69	16.22	2.35
	E-III	35.4	46.3	F9	27.24-53.17	40.48	12.5	3.39	61.17	10.53	13.47	8.39	6.87

**Fig 1:**
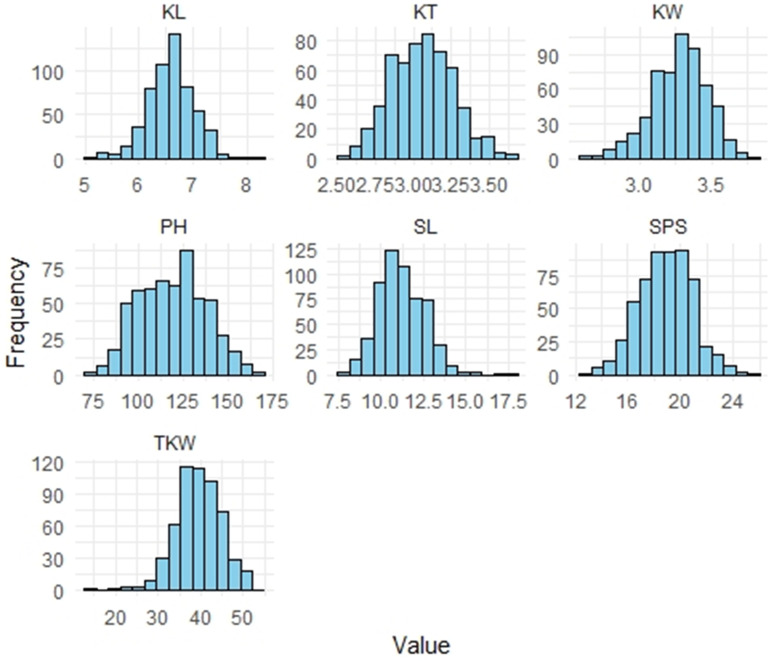
Histogram of frequency distributions for (a) Plant height (PH), (b) Spike length (SL), (c) Spikelet per spike (SLS) (d) Kernel Length (KL) (e) Kernel Weight (KW) (f) Kernel Thickness (KT), (g) Thousand kernel weight (TKW) in the RILs grown at ICAR-IARI across the years.

The distribution of agro-morphological and kernel traits showed continuous and near-normal patterns, as illustrated by frequency distribution histograms (Fig 1), supporting the quantitative nature of these traits. Variation in kernel traits including thousand TKW, KL, KW, and KT was evaluated in advanced generations. The RIL population displayed a wide range of values for all these parameters. Specifically, KL ranged from 5.13 to 8.27 mm, KW from 2.26 to 3.98 mm, KT from 2.55 to 3.66 mm, and TKW varied from 21.12 to 53.17 g across the tested environments. Synthetic 46 exhibited higher values for TKW, KL, and KT compared to HD2932, reaffirming its potential as a donor parent for grain quality improvement.

**Fig 2:**
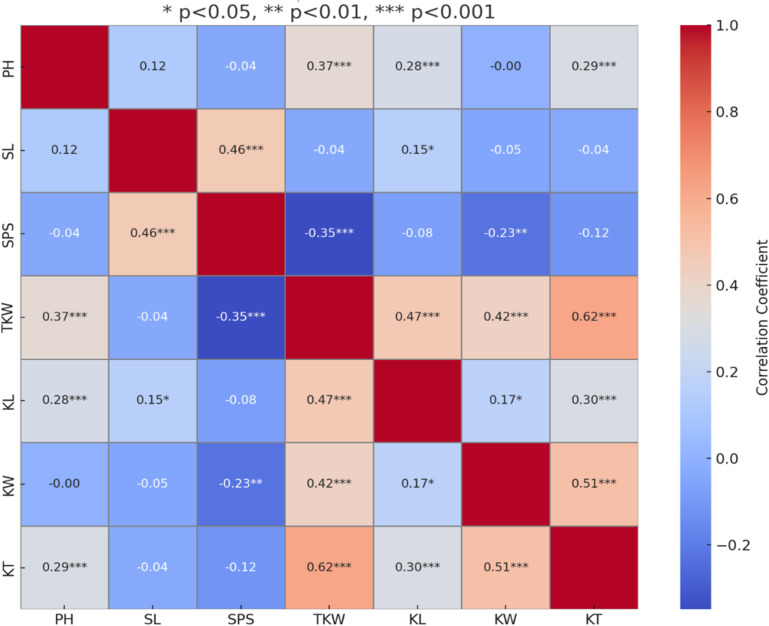
Genetic correlation coefficients among agronomic traits [plant height (PH), spike length (SL), spikelets per spike (SPS)] and kernel traits [thousand kernel weight (TKW), kernel length (KL), kernel width (KW), kernel thickness (KT)] in the RIL population evaluated at ICAR-IARI during 2021–2024. Significant values at *p < 0.05, **p < 0.01 and *** p < 0.001.

Heritability estimates for all kernel traits ranged from moderate to high across environments, reflecting stable genetic expression. The highest GCV was recorded for KL, KT, and TKW in the E-I generation, whereas KW displayed the highest GCV in E-III. Maximum PCV values for KL and KT occurred in E-II, while TKW and KW showed highest PCV values in E-II and E-III, respectively. Notably, the effect of environment on trait expression was minimal across all kernel traits, with the exception of TKW, which showed a significant environmental influence. The highest and lowest ECV values for TKW were recorded in the E-II and E-I generations, respectively. Pearson correlation analysis revealed important trait interrelationships (Fig 2). Plant height (PH) was positively and significantly correlated with TKW, KL, and KT, suggesting taller plants tended to produce heavier and larger grains. Spike length (SL) was positively correlated with both SPS and KL, indicating longer spikes may contribute to greater grain size. Conversely, SPS showed a significant negative correlation with TKW and KW, suggesting a trade-off between spikelet number and individual grain mass and width. TKW was positively and significantly correlated with KL, KW, and KT, highlighting the role of all three kernel size components in determining grain weight. KL also showed a strong positive association with KW and KT, while KT was positively and significantly correlated with TKW, KL, and KW, reinforcing the interconnected nature of grain morphology traits. Some correlations with low coefficients (e.g., r < 0.3) were statistically significant due to the relatively large sample size. These weak associations may have limited biological relevance, and their practical importance should be interpreted cautiously.

### Quantitative Trait Locus (QTL) Mapping

**Fig 3:**
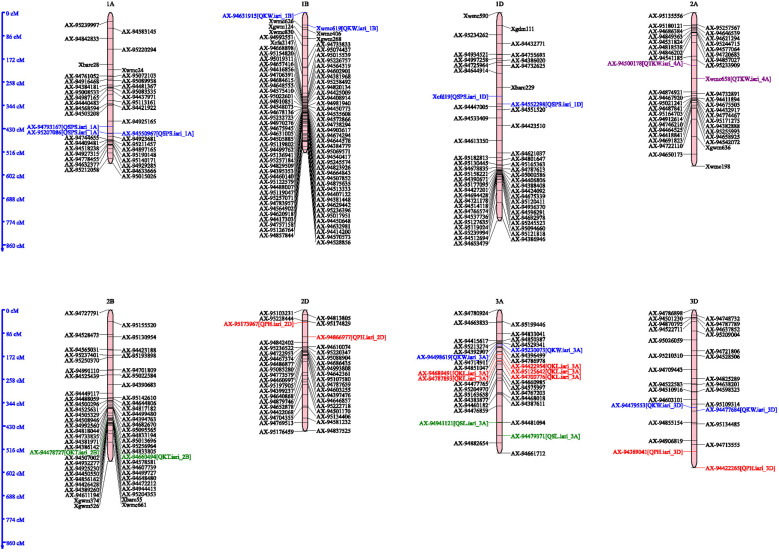
Genetic linkage map illustrating the locations of QTLs detected in the A, B, and D genomes of the RIL population derived from the cross HD2932 × Synthetic 46. QTLs were identified for plant height (PH), spike length (SL), spikelets per spike (SPS), thousand kernel weight (TKW), kernel length (KL), kernel width (KW), and kernel thickness (KT) on chromosomes 1A, 1B, 2A, 2B, 2D, 3A, and 3D.

**Fig 4:**
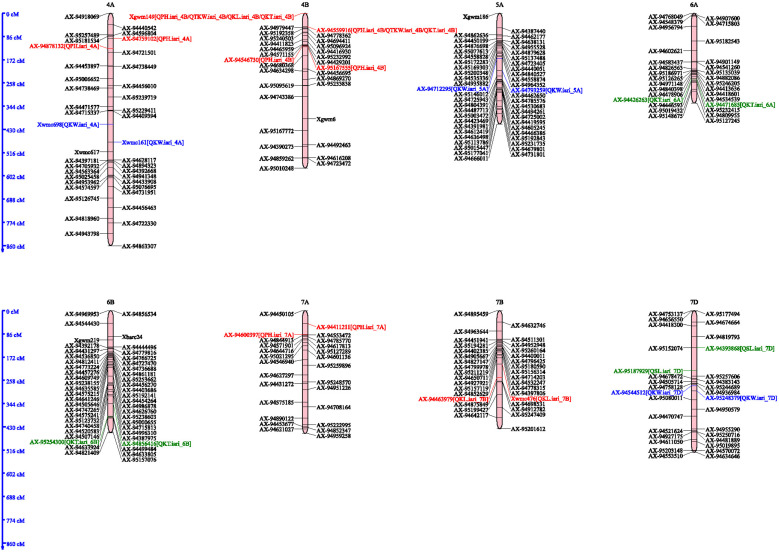
Genetic linkage map showing the QTL locations identified in the A, B, and D genomes of the RIL population developed from the cross between HD2932 and Synthetic 46. QTLs associated with plant height (PH), spike length (SL), spikelets per spike (SPS), thousand kernel weight (TKW), kernel length (KL), kernel width (KW), and kernel thickness (KT) were mapped on chromosomes 4A, 4B, 5A, 6A, 6B, 7A, 7B, and 7D.

A total of six QTLs were identified for PH on 2D, 3D, 4A, 4B and 7A chromosomes, with the LOD score ranged from 2.76–6.12 and phenotypic variance from 3.17–17.12, respectively (Table 2; Figs 3 and 4). QTL, *QPh.iari_3D* was identified on chromosome 3D and was flanked by the markers *AX-94389041* and *AX-94422265.* It had a LOD score of 4.38, which indicates strong evidence for the presence of QTL. It explained 17.122% of the phenotypic variation and had an additive effect of −7.8472. The confidence interval for this QTL ranges from 543.5 to 583 cM, indicating the region on the chromosome where the QTL is likely to be located. Two QTLs namely *QPh.iari_4B* and *Ph.iari_4B.1* were found to be located on 4B chromosome*. QPh.iari_4B* was identified in 2021–22, 2022–23, and across years. It was flanked by the marker *Xgwm149* and *AX-94559916* with LOD scores of 3.3093 to 6.1244, with the highest LOD score observed in the year 2022–23. The additive effect ranged from 3.65 to 10.96, with the highest additive effect observed in 2022–23. The confidence intervals for these QTLs vary between 0 to 21.5 cM. Another QTL, *QPh.iari_4B.1* was located between the markers *AX-94546730* and *AX-95167555* with LOD of 2.78, PVE of 3.16% spanning in the confidence interval of 120.5–124.5 cM. Similarly, a QTL related to Plant height, *QPh.iari_7A* was located on chromosome 7A, flanking between the markers *AX-94411211* and *AX-94600397*. It was an important QTL with PVE of 5.66% and the additive effect of −7.329, and the confidence interval between 50.5–80.5 cM. Another QTL for plant height, *QPh.iari_2D* was located on chromosome 2D with the flanking markers *AX-95173967* and *AX-94866977*. The LOD score for this QTL was 4.1704, and it explained the phenotypic variance of 8.81% with additive effect −9.0461. Lastly, *QPh.iari_4A*, This QTL is located on chromosome 4A with flanking markers *AX-94759102* and *AX-94878132* identified in E-III and in E-IV. With the LOD score of 2.7–4.5 it explained 3.9–6.27% of the phenotypic variance. The additive effect of this QTL is 7.63, and the confidence interval between 88.5–112.5 cM.

**Table 2:** List of QTLs identified for Agro-morphological traits such as Plant Height (PH), Spike Length (SL), Spikelet Per Spike (SPS).

Trait	Name of QTL	Envir.	Chr.	Position	Interval Markers	LOD	PVE (%)	Add.	CI
PH	*QPh.iari_3D*	E-I	3D	560	*AX-94389041-AX-94422265*	4.38	17.122	−7.8472	543.5-583
	*QPh.iari_4B*	E-I	4B	0	*Xgwm149-AX-94559916*	3.30	3.6858	3.6509	0-19.5
		E -II		13	*Xgwm149-AX-94559916*	6.1244	12.7281	10.9583	0-21.5
		E-IV		14	*Xgwm149-AX-94559916*	6.0422	13.0305	9.363	3.5-21.5
	*QPh.iari_4B.1*	E -I	4B	123	*AX-94546730-AX-95167555*	2.7884	3.1699	3.3767	120.5-124.5
	*QPh.iari_7A*	E -II	7A	67	*AX-94411211-AX-94600397*	2.7568	5.66	−7.329	50.5-80.5
	*QPh.iari_2D*	E -III	2D	66	*AX-95173967-AX-94866977*	4.1704	8.8114	−9.0461	48.5-78.5
	*QPh.iari_4A*	E -III	4A	103	*AX-94759102-AX-94878132*	4.5048	6.2728	7.6329	88.5-112.5
		E-IV		105	*AX-94759102-AX-94878132*	2.7645	3.9358	5.1443	92.5-117.5
SL	*QSl.iari_3A*	E -I	3A	445	*AX-94941121-AX-94479371*	2.7277	3.1825	0.7004	431.5-468.5
	*QSl.iari_7D*	E -I	7D	169	*AX-94393868-AX-95187929*	2.5753	5.5492	−0.9249	146.5-186.5
SPS	*QSps.iari_1A*	E -I	1A	445	*AX-94793167-AX-94550967*	3.4765	7.1414	−0.6521	434.5-447.5
	*QSps.iari_1A.1*	E-IV	1A	446	*AX-94550967-AX-95207086*	3.1109	7.4252	−0.4725	436.5-447.5
	*QSps.iari_1D*	E -I	1D	310	*Xcfd19-AX-94552298*	3.1318	6.1812	0.6069	294.5-324.5

Note: Envir: Environment, Chr: Chromosome, PVE%: Phenotypic variance (in %), add.: Additive effect, CI: Confidence interval. E-I, E-II, E-III corresponds to 3 seasons and E-IV corresponds to the pooled across the seasons

Two QTL *QSl.iari_3A* and *QTL QSl.iari_7D* were identified for Spike length, on 3A and 7D chromosomes (Table 2; Figs 3 and 4). Out of these two QTLs *QSl.iari_3A* was identified on chromosome 3A during the 2021–2022 study period. This locus was detected at a specific map location 445 (cM), with flanking markers *AX-94941121 and AX-94479371*. It accounted for approximately 3.18% of the phenotypic variance. Furthermore, its additive effect was quantified as 0.70. The confidence interval was determined to be between 431.5 and 468.5 cM. Subsequently, the QTL *QSl.iari_7D* was detected on chromosome 7D at the 169 cM point during the same cropping season of 2021–22. This locus was flanked by markers *AX-94393868 and AX-95187929*. The LOD score for this QTL was noted to be 2.5753. *QSl.iari_7D* was responsible for approximately 5.55% of the total phenotypic variance. The confidence interval for the position of this QTL spanned from 146.5 to 186.5 cM. A total of three QTLs were identified for the trait Spikelet per spike (SPS) on 1A and 1D chromosomes with the LOD and phenotypic variance ranging from 3.1–3.4 and 6.18–7.43, respectively (Table 2). The QTL *QSps.iari_1A.1* was identified in the 2021–22 environments on chromosome 1A at the 445 cM position. It was flanked by markers *AX-94793167* and *AX-94550967*. It had a LOD score of 3.4765 and accounted for 7.14% of the total phenotypic variance. The confidence interval was between 434.5 and 447.5 cM. Another QTL, *QSps.iari_1A.2*, was detected in polled data across the season. It was located on chromosome 1A at position 446 cM, with flanking markers *AX-94550967* and *AX-95207086*. It had a LOD score of 3.11 and contributed 7.43% of the total phenotypic variance (PVE). The confidence interval ranged from 436.5 to 447.5 cM. Lastly, the QTL *QSps.iari_1D* was identified in the 2021–22 cropping season. It was located on chromosome 1D at the 310 cM position, with flanking markers *Xcfd19* and *AX-94552298*. It achieved a LOD score of 3.13 and accounted for 6.18% of the phenotypic variance. The confidence interval for this QTL was between 294.5 and 324.5 cM.

A total of five QTLs were identified on 1B, 3A, 4B, and 7B chromosomes for kernel length (KL) (Table 3; Figs 3 and 4). These QTLs ranged from 2.53–4.45 and 2.39–9.07 for LOD score and phenotypic variance respectively. One of these QTLs *QKl.iari_3A* was located on chromosome 3A at position 161 cM in the 2021−22, 2023−24 and across the environments. The markers flanking this QTL were *AX-94422954* and *AX-94689491* with a LOD score of 2.55, explaining about 6.32% of the total phenotypic variance. The confidence interval for this QTL’s location was between 156.5 and 167.5 cM. Similar patterns were observed for this QTL during 2023−24 and across environments albeit with slightly varying LOD scores, PVE percentages, and additive effects. *QKl.iari_4B* was another QTL identified, located on chromosome 4B at position 0 cM in the 2021−22, 2022−23 and across years. This QTL was flanked by markers *Xgwm149* and *AX-94559916*. The LOD score was 2.6867, with the QTL accounting for approximately 6.06% of the phenotypic variance. The QTL, *QKl.iari_7B* was found on chromosome 7B at position 201cM in the 2022−23 and across years, flanked by markers *Xwmc476* and *AX-94463979*. It had a LOD score of 2.546 and accounted for about 5.69% of the phenotypic variance. *QKl.iari_3A.1* and *QKl.iari_3A.2* were detected on chromosome 3A at positions 190 and 188 cM, respectively. Each of these QTLs exhibited unique LOD scores, PVE percentages, and additive effects, with the latter implying a positive influence on the trait. *QKl.iari_1B* was identified on chromosome 1B at position 35 cM in the cropping season 2022−23 environment. This QTL, flanked by markers *AX-94631915* and *Xwmc619*, had a LOD score of 2.525 and accounted for approximately 5.52% of the phenotypic variance. When studied in the 2023−24 environment, the position shifted to 45 cM, with slight changes in the LOD score, PVE, and additive effect.

**Table 3:** List of QTLs identified for kernel quality traits, Thousand kernel weight (TKW), kernel length (KL), kernel width (KW), and kernel thickness (KT).

Trait	Name of QTL	Envir.	Chr.	Position	Interval Markers	LOD	PVE (%)	Add.	CI
TKW	*QTkw.iari_4B*	E-I	4B	0	*Xgwm149-AX-94559916*	3.2443	3.9514	1.5771	0-13.5
		E-III		0	*Xgwm149-AX-94559916*	5.6115	13.4027	1.7524	0-12.5
		E-IV		0	*Xgwm149-AX-94559916*	4.3741	10.8535	1.4167	0-12.5
	*QTkw.iari_2A*	E-I	2A	215	*AX-94500178-Xwmc658*	2.9095	12.1103	2.7559	199.5-231.5
KL	*QKl.iari_3A.1*	E-I	3A	161	*AX-94422954-AX-94689491*	2.5462	6.3233	−0.1199	156.5-167.5
		E-III		162	*AX-94422954-AX-94689491*	4.2193	7.3079	−0.1115	156.5-170.5
		E-IV		161	*AX-94422954-AX-94689491*	4.4502	6.5668	−0.1261	156.5-169.5
	*QKl.iari_4B*	E-I	4B	0	*Xgwm149-AX-94559916*	2.6867	6.0574	0.1175	0-17.5
		E-II		0	*Xgwm149-AX-94559916*	3.56	6.6779	0.1259	0-18.5
		E-IV		0	*Xgwm149-AX-94559916*	3.0842	4.0601	0.0992	0-15.5
	*QKl.iari_7B*	E-II	7B	201	*Xwmc476-AX-94463979*	2.5463	5.6916	0.1162	185.5-219.5
		E-IV		213	*Xwmc476-AX-94463979*	2.6091	9.0656	0.1479	190.5-228.5
	*QKl.iari_3A.3*	E-III	3A	190	*AX-94787893-AX-94700776*	2.8257	4.6833	0.0898	182.5-191.5
	*QKl.iari_3A.2*	E-IV	3A	188	*AX-95125642-AX-94787893*	2.9844	4.1916	0.1014	182.5-191.5
KW	*QKw.iari_1B*	E-I	1B	35	*AX-94631915-Xwmc619*	2.5253	5.5231	−0.0611	19.5-51.5
		E-III		45	*AX-94631915-Xwmc619*	3.0323	2.3911	−0.056	23.5-49.5
	*QKw.iari_3A*	E-III	3A	126	*AX-95230073-AX-94498619*	3.9093	3.0345	0.0629	125.5-130.5
	*QKw.iari_3D*	E-III	3D	373	*AX-94479553-AX-94477684*	10.5225	9.6442	−0.1126	367.5-384.5
	*QKw.iari_4A*	E-III	4A	446	*Xwmc698-Xwmc161*	2.6822	13.5676	−0.1329	432.5-464.5
	*QKw.iari_5A*	E-III	5A	168	*AX-94712295-AX-94793259*	4.1526	3.3371	−0.0662	164.5-178.5
	*QKw.iari_7D*	E-IV	7D	293	*AX-94544512-AX-95248379*	2.5056	5.6975	0.0494	284.5-313.5
KT	*QKt.iari_2B*	E-I	2B	403	*AX-94478727-AX-94660494*	2.8101	6.9672	−0.051	402.5-405.5
	*QKt.iari_4B*	E-II	4B	16	*Xgwm149-AX-94559916*	6.2704	16.3858	0.1139	8.5-23.5
		E-III		13	*Xgwm149-AX-94559916*	8.2706	19.5803	0.1119	3.5-21.5
		E-IV		15	*Xgwm149-AX-94559916*	7.8843	14.0805	0.1031	6.5-21.5
	*QKt.iari_6A*	E-II		246	*AX-94426263-AX-94471685*	3.6018	2.7845	−0.047	245.5-250.5
		E-III	6A	247	*AX-94426263-AX-94471685*	3.1389	2.7618	−0.0421	245.5-250.5
	*QKt.iari_6B*	E-II	6B	379	*AX-94856416-AX-95254300*	2.6611	4.4519	0.0594	363.5-391.5

Note: Envir: Environment, Chr: Chromosome, PVE%: Phenotypic variance (in %), add.: Additive effect, CI: Confidence interval.E-I, E-II, E-III corresponds to 3 seasons and E-IV corresponds to pooled across the seasons

A total of six QTLs were identified on 1B, 3A, 3D, 4A, 5A and 7D chromosomes for kernel width (KW) (Table 3; Figs 3 and 4). These QTLs ranged from 2.51–4.45 and 3.03–13.57 for LOD score and phenotypic variance respectively. The QTL *QKw.iari_3A* was identified on chromosome 3A at the 126 cM position during 2023–24 (E3). Its flanking markers were identified as *AX-95230073* and *AX-94498619*. The LOD score, a measure of statistical confidence in the location of the QTL, was found to be 3.9093. This QTL accounted for 3.03% of the total phenotypic variance with confidence interval of 125.5 to 130.5 cM. *QKw.iari_3D*, another QTL, was located on chromosome 3D at the 373 cM position during 2023–24 (E3). The flanking markers for this QTL were *AX-94479553* and *AX-94477684*. This QTL demonstrated a relatively high LOD score of 10.5225. It accounted for 9.64% of the phenotypic variance, the confidence interval was established between 367.5 and 384.5cM. In the same environment (2023–24), the QTL *QKw.iari_4A* was identified on chromosome 4A at the 446 cM position, flanked by markers Xwmc698 and Xwmc161. The LOD score was 2.6822, and the QTL explained a notable 13.57% of the phenotypic variance. It had a confidence interval for the QTL’s location was between 432.5 and 464.5 cM. Another QTL, *QKw.iari_5A*, was located on chromosome 5A at the 168 cM position during 2023–24, flanked by markers *AX-94712295* and *AX-94793259*. It had a LOD score of 4.1526 and accounted for 3.34% of the phenotypic variance. The confidence interval for this QTL’s position ranged from 164.5 to 178.5 cM. *QKw.iari_7D* was identified on chromosome 7D at the 293 cM position in across years data. The flanking markers were *AX-94544512* and *AX-95248379*. This QTL had a LOD score of 2.5056 and explained 5.70% of the phenotypic variance. The additive effect was positive (0.0494). The confidence interval for this QTL’s position was between 284.5 and 313.5 cM.

A total of four QTLs were identified on 2B, 4B, 6A and 6B for kernel thickness (KT). These QTLs had LOD score and phenotypic variance ranged from 2.66–8.27 and 2.76–19.58%, respectively (Table 3; Figs 3 and 4). The QTL *QKt.iari_2B* was identified on chromosome 2B at the 403 cM position within the 2021−22 cropping season. The LOD score was found to be 2.8101. This QTL explained 6.97% of the total phenotypic variance, as indicated by the PVE value. The confidence interval for the QTL’s location was narrow, ranging from 402.5 to 405.5 cM. *QKt.iari_4B*, another QTL, was located on chromosome 4B at 16 cM, 13 cM, and 15 cM during 2022−23, 2023−24 and across years, respectively. This QTL demonstrated LOD scores of 6.2704, 8.2706, and 7.8843, respectively, accounted for substantial portions of the phenotypic variance (16.39%, 19.58%, and 14.08%, respectively). The confidence interval for this QTL’s location ranged from 8.5 to 23.5 cM in the 2022−23 environment, 3.5 to 21.5 cM in the 2023−24 environment, and 6.5 to 21.5 cM in data pooled across the years. On chromosome 6A, *QKt.iari_6A* was identified at the 246 cM and 247 cM positions within the 2022−23 and 2023−24 environments respectively. The LOD scores for this QTL in each environment, were 3.6018 and 3.1389, accounted for PVE 2.78% and 2.76% respectively. The confidence interval for this QTL’s position was between 245.5 and 250.5 cM in both environments. Lastly, *QKt.iari_6B* was located on chromosome 6B at the 379 cM position in the cropping season 2022−23. This QTL had a LOD score of 2.6611 and explained 4.45% of the phenotypic variance. The confidence interval for this QTL’s position ranged from 363.5 to 391.5 cM.

Two QTLs *QTkw.iari_2A* and *QTkw.iari_4B* identified for thousand kernel weight (TKW) were identified on 2A and 4B chromosomes (Table 3; Figs 3 and 4). LOD score and phenotypic variance for these QTLs Ranged from 2.91–5.61 and 3.95–13.40 respectively. The QTL *QTkw.iari_2A* was identified on chromosome 2A in the 2021−22 environments. It was located at the 215 cM position, flanked by the markers *AX-94500178-Xwmc658*. The LOD score for this QTL was 2.9095, and it accounted for 12.11% of the phenotypic variance. The confidence interval for this QTL’s location was between 199.5 and 231.5 cM. QTL *QTkw.iari_4B* was located on chromosome 4B, and was analyzed across three environments: 2021−22, 2023−24, and polled across years. Despite the variance in the environments, the QTL was situated at position 0 cM in each instance, flanked by the markers *Xgwm149-AX-94559916*. The LOD scores of this QTL were 3.24, 5.61, and 4.37 respectively in different environments. It accounted for 3.95%, 13.40%, and 10.85% of the total phenotypic variance in each respective environment. The confidence intervals for the location of this QTL ranged from 0 to 13.5 cM for 2021−22, and 0 to 12.5 cM for 2023−24 and across environments. Further, physical position of all the significant interval marker pairs were given in Supplementary Table 4 in S1 File based on IWGSC RefSeq v1.0 for detailed study of the candidate regions.

### Co-localized QTLs

A notable finding of this study was the identification of a pleiotropic QTL on chromosome 4B flanked by markers *Xgwm149–AX-94559916*, which was associated with multiple agronomic traits, including plant height (PH), thousand kernel weight (TKW), kernel length (KL), and kernel thickness (KT). This QTL region exhibited co-localization for these traits and was consistently detected across at least two environments, indicating its consistency and potential breeding utility.. For plant height, two major QTLs, *QPh.iari_4B* and *QPh.iari_4A*, were identified flanked by *Xgwm149–AX-94422265* and *AX-94759102–AX-94878132*, respectively, and were expressed in at least three environments. For kernel length, three QTLs, *QKl.iari_3A.1*, *QKl.iari_4B*, and *QKl.iari_7B* were identified across multiple environments, explaining phenotypic variances ranging from 4.06% to 9.07%. A QTL for kernel width, *QKw.iari_1B*, was detected in multiple cropping seasons, accounting for 5.52% and 2.39% of phenotypic variance in respective seasons. Additionally, two QTLs for kernel thickness, *QKt.iari_4B* and *QKt.iari_6A*, were identified on chromosomes 4B and 6A, with phenotypic variances ranging from 14.08% to 19.58% and 2.76% to 2.78%, respectively. Collectively, the genomic region between *Xgwm149–AX-94559916* emerges as a promising candidate interval, as it harbours multiple co-localized QTLs for key agronomic traits, offering a strong target for marker-assisted selection in wheat improvement.

### Putative candidate genes

A total of 28 candidate genes associated with key agro-morphological and kernel quality traits were identified based on significant marker-trait associations (Table 4). For plant height (PH), thousand kernel weight (TKW), kernel length (KL), and kernel thickness (KT), the marker AX-94559916 on chromosome 4B (650.27 Mb) was linked with two candidate genes: *TraesCS4B02G359900* encoding a pentatricopeptide repeat (PPR) protein and *TraesCS4B02G360000* encoding a BTB/POZ and MATH domain-containing protein. Additional PH-associated markers on chromosome 4A revealed nine genes involved in diverse biological processes, including argininosuccinate lyase, RNA-dependent RNA polymerase, cytochrome P450, and Ulp1 protease. For kernel length (KL), markers on chromosomes 3A and 7B identified eight genes, such as those encoding tyrosine-specific protein phosphatase, bZIP and SANT/Myb transcription factors, F-box proteins, and phospholipase D, highlighting their regulatory roles in grain development. Kernel width (KW) was associated with six genes on chromosome 1B, including kinesin-like protein KIN-14U, PSA3, and a glycosyltransferase gene (*TraesCS1B02G081900*), suggesting roles in photosynthesis and carbohydrate metabolism. Kernel thickness (KT) was linked to three genes on chromosome 6A, including those coding for anaphase-promoting complex subunit 8 and DEAD/DEAH-box helicases.

**Table 4:** List of candidate genes located in the region of identified QTLs/flanking regions of markers for Agro-morphological and kernel quality traits of wheat.

Trait	Marker	Chr. No	Position (Mb)	TraceID	Protein
PH, TKW, KL and KT	*AX-94559916*	4B	650.27	*TraesCS4B02G359900*	Pentatricopeptide repeat (PPR) genes
				*TraesCS4B02G360000*	BTB/POZ and MATH domain-containing protein 1–6
PH	*AX-94759102*	4A	147.57	*TraesCS4A02G119700*	Argininosuccinate lyase
				*TraesCS4A02G119800*	RNA-dependent RNA polymerase
				*TraesCS4A02G119900*	Tim23-like
				*TraesCS4A02G120000*	Peptide chain release factor PrfB2, chloroplastic
				*TraesCS4A02G120100*	Glycosyltransferase 34
	*AX-94878132*	4A	631.64	*TraesCS4A02G358400*	AIG1-type guanine nucleotide-binding (G) domain
				*TraesCS4A02G358100*	Ulp1 protease family, C-terminal catalytic domain
				*TraesCS4A02G358300*	Multicopper oxidase, N-terminal
				*TraesCS4A02G358200*	Cytochrome P450
KL	*AX-94422954*	3A	623.04	*TraesCS3A02G372300*	Tyrosine-specific protein phosphatases domain
				*TraesCS3A02G372400*	Basic-leucine zipper domain
				*TraesCS3A02G372500*	F-box domain
	*AX-94689491*	3A	618.16	*TraesCS3A02G368700*	SANT/Myb domain
	*AX-94463979*	7B	725.52	*TraesCS7B02G468200*	Phospholipase D
				*TraesCS7B02G468300*	Phospholipase D
				*TraesCS7B02G468400*	Rhodanese-like domain
				*TraesCS7B02G468500*	Protein coding
KW	*AX-94631915*	1B	589.06	*TraesCS1B02G360700*	Kinesin-like protein KIN-14U
				*TraesCS1B02G360500*	Photosystem I assembly factor PSA3
				*TraesCS1B02G360600*	F-box domain
				*TraesCS1B02G360400*	P-loop containing nucleoside triphosphate hydrolase
				*TraesCS1B02G360800*	Reticulon-like protein
	*Xwmc619*	1B	65.28	*TraesCS1B02G081900*	Glycosyltransferases
KT	*AX-94426263*	6A	465.93	*TraesCS6A02G251400*	Anaphase-promoting complex subunit 8
				*TraesCS6A02G251300*	Protein coding
	*AX-94471685*	6A	446.30	*TraesCS6A02G236800*	Helicase superfamily 1/2, ATP-binding domain// DEAD/DEAH box helicase domain

Note: Plant height (PH), Thousand kernel weight (TKW), Kernel length (KL), Kernel width (KW), and Kernel thickness (KT).

## Discussion

Mapping and characterization of yield and grain quality traits are critical in wheat for enhancing genetic gains and ensuring the sustainability of wheat production systems. In the current study, highest broad sense heritability was observed for PH followed by SL and SPS. Similar trends were observed for GCV and PCV whereas reverse trends were observed for ECV. The highest genetic advance was for all three traits that were observed in F_7_ followed by other generations. PH, SL and SPS tested in the RIL population tested in F_7_, F_8_ and F_9_ revealed a continuous and near-normal distribution for all studied traits which indicates the polygenic nature of the traits. The above result indicates that PH is a relatively stable trait with respect to SL and SLS. Similar results were also reported in previous studies [28].

Kernel weight and number per spike work together to affect grain yield [34,35]. TKW is not only an important element of grain production but is also frequently employed as a determinant of wheat’s economic worth. TKW is highly and positively linked with kernel size and shape, including KL, KW, and KT [35–38]. Wheat kernel weight, yield, and commercial value are all positively impacted by larger kernels [18,19,39]). TKW is regulated by factors related to kernel size, which affect wheat production; both traits are highly heritable [40–45]. In this study our focus was on investigating the variation in kernel parameters, including TKW, KL, KW, and KT, in advanced filial generations (RILs). The findings shed light on the potential application of synthetic wheat donor lines in wheat breeding programs and highlight the impact of the Synthetic wheat donor (Syn46) on recombination in kernel parameters in advanced filial generations. The highest percentage of coefficient of variation (CV) was observed for KW, KT, and TKW in the E-III, while for KL, it was found in the E-II. Moderate to high heritability was observed for all the studied traits in all environments. The highest genetic coefficient of variation (GCV) was observed for KL, KT, and TKW in the E-I, and for KW in the E-III generation. The highest phenotypic coefficient of variation (PCV) for KL and KT was observed in the E-II, while for TKW and KW, it was in the E-II and E-III generations, respectively [40,46–48].

TKW plays a crucial role in determining yield and can be directly targeted for yield improvement [49]. Identifying and confirming specific QTLs/genes related to grain size and weight could aid in enhancing yield. In this study, highly heritable traits such as TKW, KW, KL, and KT showed a positive and significant correlation across different environments, with high heritability. These findings align with previous research, indicating that grain weight is largely influenced by grain size components, such as grain length and width [46,47,50,51]. A total of six QTLs were identified for plant height (PH), namely *QPh.iari_3D, QPh.iari_4B, QPh.iari_4B.1, QPh.iari_7A, QPh.iari_2D,* and *QPh.iari_4A*, found to be located on chromosome 3D, 4A, 4B, 7A and 2D with phenotypic variance ranging from 3.17% to 17.12%. Notably, *QPh.iari_4B* and *QPh.iari_4A* were flanked byXgwm149-AX-94422265 and AX-94759102-AX94878132 respectively and identified as a major QTL, in at least three environments. QTLs for PH were also reported in previous studies on 3D [28], 4A [28], 4B [44,52], 7A [53,54], and 2D.

For spike length (SL), two QTLs, *QSl.iari_3A* and *QSl.iari_7D*, explaining 3.18% and 5.55% of phenotypic variance, respectively were identified on chromosomes 3A and 7D, explaining 3.18% and 5.55% of phenotypic variance, respectively. Previous studies also identified QTLs for spike length in various linkage groups [55,56]. Additionally, three QTLs, *QSps.iari_1A.1, QSps.iari_1A.2,* and *QSps.iari_1D*, were identified for spikelet per spike (SPS) on chromosomes 1A and 1D, with phenotypic variances of 7.14%, 7.43%, and 6.18%, respectively. QTL for SPS were identified on almost all the chromosomes in previous studies [56].

In the present study two major QTLs, *QTkw.iari_4B* and *QTkw.iari_2A*, were identified for TKW between *Xgwm149 - AX-94422265* and *AX-94500178 – Xwmc658* markers, located on chromosomes 4B and 2A, with phenotypic variances ranging from 3.95% to 13.40%. In previous studies various QTLs for TKW were identified on 2A [47,57–65] and 4B chromosomes [47,58,62,64,66–75]. For kernel length (KL), five QTLs, *QKl.iari_3A.1, QKl.iari_3A.2*, *QKl.iari_3A.3, QKl.iari_4B* and *QKl.iari_7B* identified on 3A, 4B, 7B.*QKl.iari_3A.1, QKl.iari_4B* and *QKl.iari_7B* showing presence in more than two environments and phenotypic variances ranging from 4.06% to 9.07%. QTLs were identified on chromosomes 3A [69], 4B, [67,69,76] and 7B [71,72] in previous studies.

Moreover, six QTLs, *QKw.iari_1B QKw.iari_3A, QKw.iari_3D, QKw.iari_4A, QKw.iari_5A,* and *QKw.iari_7D* were identified for KW. Among identified QTLs, two major QTLs, *QKw.iari_3D* and *QKw.iari_4A,* showed phenotypic variances of 9.7% and 13.57%, respectively. Additionally, one QTL, *QKw.iari_1B,* was detected in multiple cropping seasons, explaining 5.52% and 2.39% of phenotypic variance for the respective seasons. QTLs associated with kernel width (KW) on chromosomes were identified on 1B [64], 3A [64], 3D [57], 4A, 5A [67], and 7D [73] in previous studies. Furthermore, four QTLs, *QKt.iari_2B, QKt.iari_4B, QKt.iari_6A*, and *QKt.iari_6B,* were associated with kernel thickness (KT) on chromosomes 2B, 4B, 6A, and 6B, with phenotypic variances ranging from 14.08% to 19.58% and 2.76% to 2.78%, respectively. QTLs associated with kernel thickness (KT) in wheat were identified in previous studies by Gegas et al. [64].

A key finding of this study was the identification of a pleiotropic and co-localized QTL on chromosome 4B (Xgwm149–AX-94559916) associated with PH, TKW, KL, and KT, consistently detected across environments. Notably, *QPh.iari_4B* and *QPh.iari_4A* were detected in three environments. QTkw.iari_4B (3.95–13.40% PV) and three QTLs (*QKl.iari_3A.1*, *4B*, *7B*) were identified for KL. One KW QTL (*QKw.iari_1B*) was consistent across seasons, and KT QTLs on 4B and 6A showed high stability. These findings suggest a valuable genomic region for marker-assisted selection targeting yield and grain quality traits in bread wheat. Although several QTLs were consistently detected across years, we did not explicitly model genotype × environment (G×E) interactions. Future studies should employ multi-environment QTL mapping approaches such as MET-based ICIM or QTL × environment interaction models to achieve a more precise dissection of QTL stability across environments. While the population of 188 RILs provided adequate power for detecting major-effect QTLs with the LOD score of 2.5, the resolution for minor-effect loci may be limited. Therefore, validation of these QTLs in larger, fine maping of identified genomic region or independent populations is recommended to improve confidence and minimize the risk of false positives.

A total of 28 candidate genes were associated with agro-morphological and kernel quality traits. A major QTL identified on chromosome 4B, flanked by marker AX-94559916, was found to be associated with multiple traits including plant height (PH), thousand kernel weight (TKW), kernel length (KL), and kernel thickness (KT). This co-localized region spans the interval 650269749–650271356 bp and harbors the candidate gene *TraesCS4B02G359900*, which encodes a Pentatricopeptide repeat (PPR) protein. PPR genes are known to play critical roles in plant development and grain formation. Notably, PPR family genes such as GRMZM2G353195 and GRMZM2G141202 have been reported as key candidates linked to yield and kernel-related traits in maize [74], supporting the functional relevance of this gene in wheat as well. A QTL for plant height (PH) was located on chromosome 4A, spanning the interval 631,577,529–631,579,059 bp, and harbors the gene *TraesCS4A02G358200*, encoding a Cytochrome P450 protein. Members of the Cytochrome P450 family are known to regulate various physiological processes in plants. Specifically, *OsCYP96B4*, a Cytochrome P450 family member in rice, has been shown to reduce plant height in a transcript dosage-dependent manner [75], suggesting a potential regulatory role of this gene in plant height modulation in wheat as well. Future studies should include expression validation of the identified candidate genes within the QTL regions using approaches such as RNAseq or qPCR to provide functional evidence and strengthen their biological relevance.

## Conclusion

This study identified genomic regions associated with important agro-morphological and kernel quality traits in wheat using a RIL population derived from HD2932 × Synthetic46. Through multi season field evaluations and high-density genotyping with 910 SSR markers and a 35K SNP array, a total of 28 QTLs were mapped. Among these, a co-localized QTL region on chromosome 4B, flanked by *Xgwm149–AX-94559916*, was consistently associated with PH, TKW, KL, and KT across environments, making it a promising candidate for simultaneous trait improvement. The identification of superior RILs (RIL-122 and RIL-66) with favorable kernel traits provides immediate value for pre-breeding and variety development. Furthermore, the in-silico identification of 28 candidate genes within major QTL regions offers insights into the genetic control and potential molecular pathways influencing yield and grain quality traits in wheat. Future research should focus on functional validation of these candidate genes and the integration of consistent QTLs into marker-assisted and genomic selection pipelines to develop high-yielding, climate-resilient wheat cultivars.

## Supporting Information

S1 File Supplementary Table 1 Monthly-wise weather data for the crop season 2021–22, 2022–23, and 2023–24. **Supplementary Table 2.** ANOVA for the year 2021-22(E-I), 2022-23(E-II), and 2023-24 (E-IV) for the traits plant height (PH), spike length (SL), spikelets per spike (SPS), thousand kernel weight (TKW), kernel length (KL), kernel width (KW), and kernel thickness (KT). **Supplementary Table 3.** List of top 10 RILs for surpassing parental lines for the traits plant height (PH), spike length (SL), spikelets per spike (SPS), thousand kernel weight (TKW), kernel length (KL), kernel width (KW), and kernel thickness (KT). **Supplementary Table 4.** Physical position of interval markers represented with chromosome number and position in mb.
(DOCX)
