# Supplementary material for: High resolution mapping of agro-morphological and grain traits in bread wheat using SNP-based QTL analysis
Source: PLoS One. 2026 Jan 2;21(1):e0340263. doi: 10.1371/journal.pone.0340263 (PMC12758827; doi:10.1371/journal.pone.0340263)
Supplement: S1 File — Monthly-wise weather data for the crop season 2021–22, 2022–23, and 2023–24. Supplementary Table 2. ANOVA for the year 2021-22(E-I), 2022-23(E-II), and 2023-24 (E-IV) for the traits plant height (PH), spike length (SL), spikelets per spike (SPS), thousand kernel weight (TKW), kernel length (KL), kernel width (KW), and kernel thickness (KT). Supplementary Table 3. List of top 10 RILs for surpassing parental lines for the traits plant height (PH), spike length (SL), spikelets per spike (SPS), thousand kernel weight (TKW), kernel length (KL), kernel width (KW), and kernel thickness (KT). Supplementary Table 4. Physical position of interval markers represented with chromosome number and position in mb. (DOCX) [file pone.0340263.s001.docx]

Supplementary Table 1: Monthly-wise weather data for the crop season 2021–22, 2022–23, and 2023–24.

| Month | Year | Max.Temp (^o^C) | Min.Temp.(^o^C) | Rainfall (mm) | Relative Humidity (% | Sunshine (hrs) |
| --- | --- | --- | --- | --- | --- | --- |
| November | 2021 | 26.86 | 10.02 | 0.11 | 82.73 | 3.87 |
| December | 2021 | 24.63 | 8.29 | 0.06 | 86.08 | 4.65 |
| January | 2022 | 18.04 | 6.04 | 2.12 | 90.68 | 2.89 |
| February | 2022 | 21.96 | 7.29 | 1.25 | 89.71 | 4.77 |
| March | 2022 | 31.96 | 14.96 | 0.01 | 80.35 | 6.38 |
| April | 2022 | 37.04 | 18.26 | 0.18 | 68.03 | 8.77 |
| November | 2022 | 28.25 | 14.67 | 0.00 | 89.07 | 5.16 |
| December | 2022 | 22.59 | 6.69 | 0.00 | 87.87 | 5.27 |
| January | 2023 | 18.55 | 5.50 | 1.08 | 89.61 | 3.56 |
| February | 2023 | 27.20 | 10.26 | 0.00 | 83.14 | 7.66 |
| March | 2023 | 29.86 | 15.28 | 3.40 | 83.65 | 6.48 |
| April | 2023 | 35.08 | 18.93 | 0.39 | 73.63 | 7.98 |
| November | 2023 | 27.38 | 12.85 | 1.40 | 89.40 | 0.40 |
| December | 2023 | 23.13 | 7.36 | 0.00 | 89.30 | 0.30 |
| January | 2024 | 17.11 | 7.18 | 0.00 | 91.87 | 1.71 |
| February | 2024 | 22.77 | 8.33 | 1.00 | 87.40 | 5.51 |
| March | 2024 | 29.33 | 13.02 | 0.40 | 75.50 | 7.56 |
| April | 2024 | 36.81 | 19.45 | 0.53 | 72.47 | 8.11 |

Supplementary Table 2: ANOVA for the year 2021-22(E-I), 2022-23(E-II), and 2023-24 (E-IV) for the traits plant height (PH), spike length (SL), spikelets per spike (SPS), thousand kernel weight (TKW), kernel length (KL), kernel width (KW), and kernel thickness (KT).

|  |  |  | E-I |  |  |  |  |  |
| --- | --- | --- | --- | --- | --- | --- | --- | --- |
| Source | D.F. | PH | SL | SPS | TKW | KL | KW | KT |
| Treatment | 189 | 394.18^**^ | 3.62^**^ | 10.60^**^ | 64.87^**^ | 0.48^**^ | 0.06^**^ | 0.08^**^ |
| Replication | 1 | 83.90^**^ | 1.22^*^ | 1.28 | 30.23^**^ | 0.93^**^ | 0.04 | 0.09^*^ |
| Error | 189 | 6.42 | 0.20 | 1.37 | 2.02 | 0.12 | 0.02 | 0.02 |
| TOTAL | 379 |  |  |  |  |  |  |  |
|  |  |  | E-II |  |  |  |  |  |
| Source | D.F. | PH | SL | SPS | TKW | KL | KW | KT |
| Treatment | 189 | 555.85^**^ | 3.32^**^ | 10.17^**^ | 59.19^*^ | 0.40^**^ | 0.06^**^ | 0.06^**^ |
| Replication | 1 | 1632.40^**^ | 3.36 | 22.60^**^ | 49.61 | 1.01^**^ | 0.04 | 0.21^**^ |
| Error | 189 | 65.67 | 1.05 | 3.19 | 42.89 | 0.05 | 0.02 | 0.01 |
| TOTAL | 379 |  |  |  |  |  |  |  |
|  |  |  | E-III |  |  |  |  |  |
| Source | D.F. | PH | SL | SPS | TKW | KL | KW | KT |
| Treatment | 189 | 495.31^**^ | 131.59 | 4.88^**^ | 47.94^**^ | 0.24^**^ | 0.10^**^ | 0.06^**^ |
| Replication | 1 | 4058.07^**^ | 92.27 | 12.24 | 37.77 | 3.94^**^ | 0.25^**^ | 0.23^**^ |
| Error | 189 | 85.49 | 128.15 | 3.45 | 11.55 | 0.08 | 0.02 | 0.01 |
| TOTAL | 379 |  |  |  |  |  |  |  |

^**^Significance at 1 % level of significance, ^*^Significance at 5 % level of significance

Supplementary Table 3: List of top 10 RILs for surpassing parental lines for the traits plant height (PH), spike length (SL), spikelets per spike (SPS), thousand kernel weight (TKW), kernel length (KL), kernel width (KW), and kernel thickness (KT).

| **E-I** | | | | | | | | | | | | | | | | | | | | |
| --- | --- | --- | --- | --- | --- | --- | --- | --- | --- | --- | --- | --- | --- | --- | --- | --- | --- | --- | --- | --- |
| **RILs** | **PH** | **%change** | **RILs** | **SL** | **%change** | **RILs** | **SPS** | **%change** | **RILs** | **KL** | **%change** | **RILs** | **KW** | **%change** | **RILs** | **KT** | **%change** | **RILs** | **TKW** | **%change** |
| HD 2932 | 97.5 |  | HD 2932 | 10.15 |  | HD 2932 | 20.01 |  | HD 2932 | 6.276 |  | HD 2932 | 3.5 |  | HD 2932 | 2.88 |  | HD 2932 | 34.005 |  |
| Syn 46 | 117.2 |  | Syn 46 | 11.45 |  | Syn 46 | 17.66 |  | Syn 46 | 8.237 |  | Syn 46 | 2.98 |  | Syn 46 | 3.6 |  | Syn 46 | 43 |  |
| RIL-73 | 73.3 | -24.8 | RIL-82 | 15.25 | 33.2 | RIL-127 | 25.76 | 28.7 | Syn 46 | 8.237 | 0 | RIL-101 | 3.62 | 3.4 | RIL-77 | 3.66 | 1.7 | RIL-40 | 51.795 | 20.5 |
| RIL-7 | 74 | -24.1 | RIL-39 | 14.3 | 24.9 | RIL-81 | 24.26 | 21.2 | RIL-48 | 7.909 | -4 | RIL-65 | 3.56 | 1.7 | RIL-104 | 3.65 | 1.4 | RIL-16 | 51.05 | 18.7 |
| RIL-52 | 77.7 | -20.3 | RIL-97 | 14.15 | 23.6 | RIL-128 | 23.75 | 18.7 | RIL-43 | 7.888 | -4.2 | RIL-25 | 3.56 | 1.7 | RIL-101 | 3.64 | 1.1 | RIL-31 | 49.835 | 15.9 |
| RIL-109 | 80 | -17.9 | RIL-35 | 14.05 | 22.7 | RIL-180 | 23.64 | 18.1 | RIL-122 | 7.675 | -6.8 | RIL-87 | 3.54 | 1.1 | Syn 46 | 3.6 | 0 | RIL-122 | 49.43 | 15 |
| RIL-79 | 82.2 | -15.7 | RIL-80 | 13.75 | 20.1 | RIL-74 | 23.63 | 18.1 | RIL-74 | 7.636 | -7.3 | RIL-28 | 3.54 | 1.1 | RIL-25 | 3.54 | -1.7 | RIL-126 | 48.045 | 11.7 |
| RIL-28 | 82.3 | -15.6 | RIL-9 | 13.7 | 19.7 | RIL-6 | 23.27 | 16.3 | RIL-34 | 7.601 | -7.7 | RIL-2 | 3.53 | 0.9 | RIL-55 | 3.53 | -1.9 | RIL-32 | 47.035 | 9.4 |
| RIL-1 | 83 | -14.9 | RIL-46 | 13.6 | 18.8 | RIL-155 | 22.98 | 14.8 | RIL-178 | 7.535 | -8.5 | RIL-55 | 3.51 | 0.3 | RIL-92 | 3.53 | -1.9 | RIL-101 | 46.015 | 7 |
| RIL-20 | 83.9 | -13.9 | RIL-4 | 13.6 | 18.8 | RIL-91 | 22.84 | 14.1 | RIL-58 | 7.452 | -9.5 | RIL-3 | 3.51 | 0.3 | RIL-112 | 3.52 | -2.2 | RIL-49 | 45.88 | 6.7 |
| RIL-90 | 84 | -13.8 | RIL-18 | 13.6 | 18.8 | RIL-103 | 22.72 | 13.5 | RIL-164 | 7.408 | -10.1 | HD 2932 | 3.5 | 0 | RIL-86 | 3.52 | -2.2 | RIL-104 | 45.56 | 6 |
| RIL-154 | 84.2 | -13.6 | RIL-11 | 13.55 | 18.3 | RIL-92 | 22.51 | 12.5 | RIL-148 | 7.362 | -10.6 | RIL-96 | 3.5 | 0 | RIL-75 | 3.5 | -2.8 | RIL-86 | 45.37 | 5.5 |
| **E-II** | | | | | | | | | | | | | | | | | | | | |
| HD 2932 | 102.7 |  | HD 2932 | 10.11 |  | HD 2932 | 19.67 |  | HD 2932 | 6.17 |  | HD 2932 | 3.5 |  | HD 2932 | 2.68 |  | HD 2932 | 34.93 |  |
| Syn 46 | 137.1 |  | Syn 46 | 12.06 |  | Syn 46 | 17.33 |  | Syn 46 | 7.21 |  | Syn 46 | 3.04 |  | Syn 46 | 3.26 |  | Syn 46 | 46.15 |  |
| RIL-52 | 80.5 | -21.6 | RIL-166 | 15.52 | 28.7 | RIL-127 | 25 | 27.1 | RIL-104 | 7.5 | 4 | RIL-55 | 3.68 | 5.1 | RIL-156 | 3.41 | 4.6 | RIL-22 | 51.35 | 11.3 |
| RIL-91 | 87.6 | -14.7 | RIL-91 | 14.83 | 23 | RIL-91 | 24 | 22 | RIL-139 | 7.41 | 2.8 | RIL-4 | 3.68 | 5.1 | RIL-150 | 3.38 | 3.7 | RIL-16 | 51.2 | 10.9 |
| RIL-90 | 89 | -13.3 | RIL-179 | 14.63 | 21.3 | RIL-180 | 23.67 | 20.3 | RIL-122 | 7.39 | 2.5 | RIL-65 | 3.61 | 3.1 | RIL-111 | 3.35 | 2.8 | RIL-49 | 51.2 | 10.9 |
| RIL-19 | 89.1 | -13.2 | RIL-6 | 14.25 | 18.2 | RIL-183 | 23 | 16.9 | RIL-81 | 7.34 | 1.8 | RIL-122 | 3.59 | 2.6 | RIL-101 | 3.33 | 2.1 | RIL-17 | 51.18 | 10.9 |
| RIL-117 | 89.2 | -13.1 | RIL-175 | 14.05 | 16.5 | RIL-128 | 23 | 16.9 | RIL-166 | 7.33 | 1.7 | RIL-131 | 3.59 | 2.6 | RIL-151 | 3.32 | 1.8 | RIL-32 | 50.2 | 8.8 |
| RIL-6 | 89.3 | -13 | RIL-146 | 13.92 | 15.4 | RIL-6 | 22.67 | 15.3 | RIL-88 | 7.32 | 1.5 | RIL-43 | 3.59 | 2.6 | RIL-66 | 3.31 | 1.5 | RIL-26 | 50.08 | 8.5 |
| RIL-83 | 90.4 | -12 | RIL-62 | 13.92 | 15.4 | RIL-62 | 22.67 | 15.3 | RIL-62 | 7.31 | 1.4 | RIL-66 | 3.59 | 2.6 | RIL-12 | 3.31 | 1.5 | RIL-66 | 49.75 | 7.8 |
| RIL-155 | 91 | -11.4 | RIL-127 | 13.83 | 14.7 | RIL-136 | 22.67 | 15.3 | RIL-148 | 7.31 | 1.4 | RIL-96 | 3.58 | 2.3 | RIL-119 | 3.3 | 1.2 | RIL-127 | 49.23 | 6.7 |
| RIL-118 | 92.4 | -10 | RIL-123 | 13.79 | 14.3 | RIL-81 | 22.67 | 15.3 | RIL-11 | 7.3 | 1.2 | RIL-2 | 3.56 | 1.7 | RIL-165 | 3.29 | 0.9 | RIL-178 | 49.2 | 6.6 |
| RIL-79 | 94.4 | -8.1 | RIL-8 | 13.67 | 13.3 | RIL-133 | 22 | 11.8 | RIL-67 | 7.3 | 1.2 | RIL-101 | 3.56 | 1.7 | RIL-115 | 3.29 | 0.9 | RIL-78 | 48.9 | 6 |
| **E-III** | | | | | | | | | | | | | | | | | | | | |
| HD 2932 | 99 |  | HD 2932 | 9.56 |  | HD 2932 | 20.7 |  | HD 2932 | 6.07 |  | HD 2932 | 3.35 |  | HD 2932 | 2.84 |  | HD 2932 | 35.43 |  |
| Syn 46 | 127.1 |  | Syn 46 | 10.23 |  | Syn 46 | 18.3 |  | Syn 46 | 7.07 |  | Syn 46 | 3.01 |  | Syn 46 | 3.38 |  | Syn 46 | 46.27 |  |
| RIL-73 | 91.8 | -7.3 | RIL-113 | 17.69 | 72.9 | RIL-71 | 23.6 | 14 | RIL-148 | 7.26 | 2.7 | RIL-112.2 | 3.8 | 13.4 | RIL-122 | 3.39 | 0.3 | RIL-32 | 53.17 | 14.9 |
| RIL-118 | 95.1 | -3.9 | RIL-28 | 17.22 | 68.3 | RIL-128 | 22.9 | 10.6 | RIL-178 | 7.26 | 2.7 | RIL-152.2 | 3.68 | 9.9 | Syn 46 | 3.38 | 0 | RIL-66 | 52.01 | 12.4 |
| RIL-28 | 96.4 | -2.6 | RIL-166 | 13.72 | 34.1 | RIL-135 | 22.8 | 10.1 | RIL-58 | 7.2 | 1.8 | RIL-110.1 | 3.68 | 9.9 | RIL-106 | 3.34 | -1.2 | RIL-77 | 51.41 | 11.1 |
| RIL-90 | 96.6 | -2.4 | RIL-175 | 13.51 | 32.1 | RIL-81 | 22.7 | 9.7 | RIL-1 | 7.17 | 1.4 | RIL-135.3 | 3.67 | 9.6 | RIL-153 | 3.33 | -1.5 | RIL-122 | 51.17 | 10.6 |
| RIL-59 | 96.6 | -2.4 | RIL-74 | 12.97 | 26.8 | RIL-169 | 22.4 | 8.2 | RIL-74 | 7.15 | 1.1 | RIL-117.6 | 3.66 | 9.3 | RIL-11 | 3.3 | -2.4 | RIL-22 | 51.05 | 10.3 |
| HD 2932 | 99 | 0 | RIL-103 | 12.91 | 26.2 | RIL-89 | 22.4 | 8.2 | RIL-168 | 7.15 | 1.1 | RIL-113.8 | 3.65 | 9 | RIL-22 | 3.27 | -3.3 | RIL-40 | 50.37 | 8.9 |
| RIL-173 | 101.6 | 2.6 | RIL-111 | 12.78 | 24.9 | RIL-141 | 22.1 | 6.8 | RIL-88 | 7.15 | 1.1 | RIL-110 | 3.65 | 9 | RIL-164 | 3.27 | -3.3 | RIL-162 | 49.78 | 7.6 |
| RIL-114 | 101.8 | 2.8 | RIL-99 | 12.71 | 24.2 | RIL-5 | 22.1 | 6.8 | RIL-5 | 7.12 | 0.7 | RIL-107.2 | 3.64 | 8.7 | RIL-16 | 3.26 | -3.6 | RIL-43 | 49.56 | 7.1 |
| RIL-102 | 102.6 | 3.6 | RIL-135 | 12.71 | 24.2 | RIL-106 | 21.7 | 4.8 | RIL-11 | 7.11 | 0.6 | RIL-102.6 | 3.63 | 8.4 | RIL-154 | 3.25 | -3.8 | RIL-5 | 49.52 | 7 |
| RIL-20 | 103.4 | 4.4 | RIL-141 | 12.69 | 24 | RIL-64 | 21.6 | 4.3 | RIL-34 | 7.11 | 0.6 | RIL-121.7 | 3.62 | 8.1 | RIL-78 | 3.25 | -3.8 | RIL-102 | 49.06 | 6 |

Supplementary Table 4: Physical position of interval markers represented with chromosome number and position in mb

| Interval Markers | Chromosome | Position in mb of left marker | Position in mb of right marker |
| --- | --- | --- | --- |
| *Xgwm149-AX-94559916* | 4B | 544649887 | 650273959 |
| *AX-94389041-AX-94422265* | 3D | 128542788 | 304404395 |
| *AX-94393868-AX-95187929* | 7D | 620141995 | 577595913 |
| *AX-94411211-AX-94600397* | 7A | 663961824 | 6760340 |
| *AX-94422954-AX-94689491* | 3A | 623038468 | 618164627 |
| *AX-94426263-AX-94471685* | 6A | 465934084 | 446301734 |
| *AX-94478727-AX-94660494* | 2B | 713697676 | 697511730 |
| *AX-94479553-AX-94477684* | 3D | 566484097 | 606890308 |
| *AX-94500178-Xwmc658* | 2A | 716457923 | 771166661 |
| *AX-94544512-AX-95248379* | 7D | 80974297 | 69956477 |
| *AX-94546730-AX-95167555* | 4B | 46609742 | 66811553 |
| *AX-94550967-AX-95207086* | 1A | 3381102 | 8297044 |
| *AX-94631915-Xwmc619* | 1B | 589061178 | 65282680 |
| *AX-94712295-AX-94793259* | 5A | 14844490 | 702136468 |
| *AX-94759102-AX-94878132* | 4A | 147565459 | 631642603 |
| *AX-94787893-AX-94700776* | 3A | 517662617 | 483921467 |
| *AX-94793167-AX-94550967* | 1A | 550970059 | 3381102 |
| *AX-94856416-AX-95254300* | 6B | 4174022 | 710150091 |
| *AX-94941121-AX-94479371* | 3A | 611702554 | 614231054 |
| *AX-95125642-AX-94787893* | 3A | 571407671 | 517662617 |
| *AX-95173967-AX-94866977* | 2D | 431674835 | 11393132 |
| *AX-95230073-AX-94498619* | 3A | 700564359 | 691106993 |
| *Xcfd19-AX-94552298* | 1D | 329723587 | 368340070 |
| *Xwmc476-AX-94463979* | 7B | 359039715 | 725520291 |
| *Xwmc698-Xwmc161* | 4A | 621809872 | 625368161 |
